# Supplementary material for: Murine blastocysts generated by in vitro fertilization show increased Warburg metabolism and altered lactate production
Source: eLife. 2022 Sep 15;11:e79153. doi: 10.7554/eLife.79153 (PMC9519152; doi:10.7554/eLife.79153)
Supplement: Figure 7—source data 1. — The value of each biological replicate was the average of 4 technical replicates. (i) Muscle, Western blot images (ppt), source data (xls). (ii) Fat, Western blot images (ppt), source data (xls). (iii) Liver, Western blot images (ppt), source data (xls). [file elife-79153-fig7-data1.zip › Figure 7. Fat soruce images .pptx]

## Slide 1
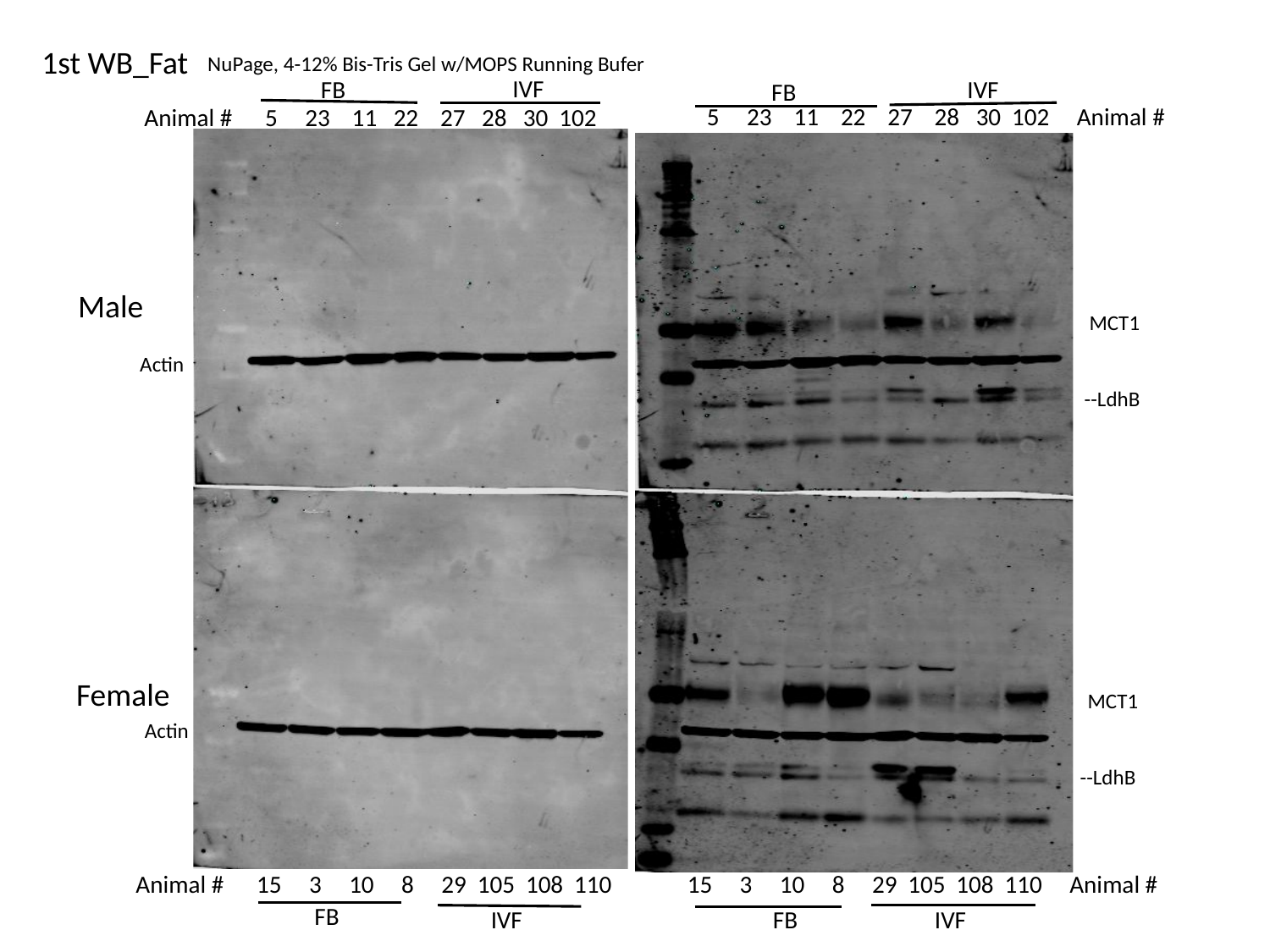

1st WB_Fat
NuPage, 4-12% Bis-Tris Gel w/MOPS Running Bufer
IVF
FB
IVF
FB
5 23 11 22 27 28 30 102 Animal #
Animal # 5 23 11 22 27 28 30 102
Male
MCT1
Actin
--LdhB
Female
MCT1
Actin
--LdhB
Animal # 15 3 10 8 29 105 108 110
15 3 10 8 29 105 108 110 Animal #
FB
IVF
IVF
FB

## Slide 2
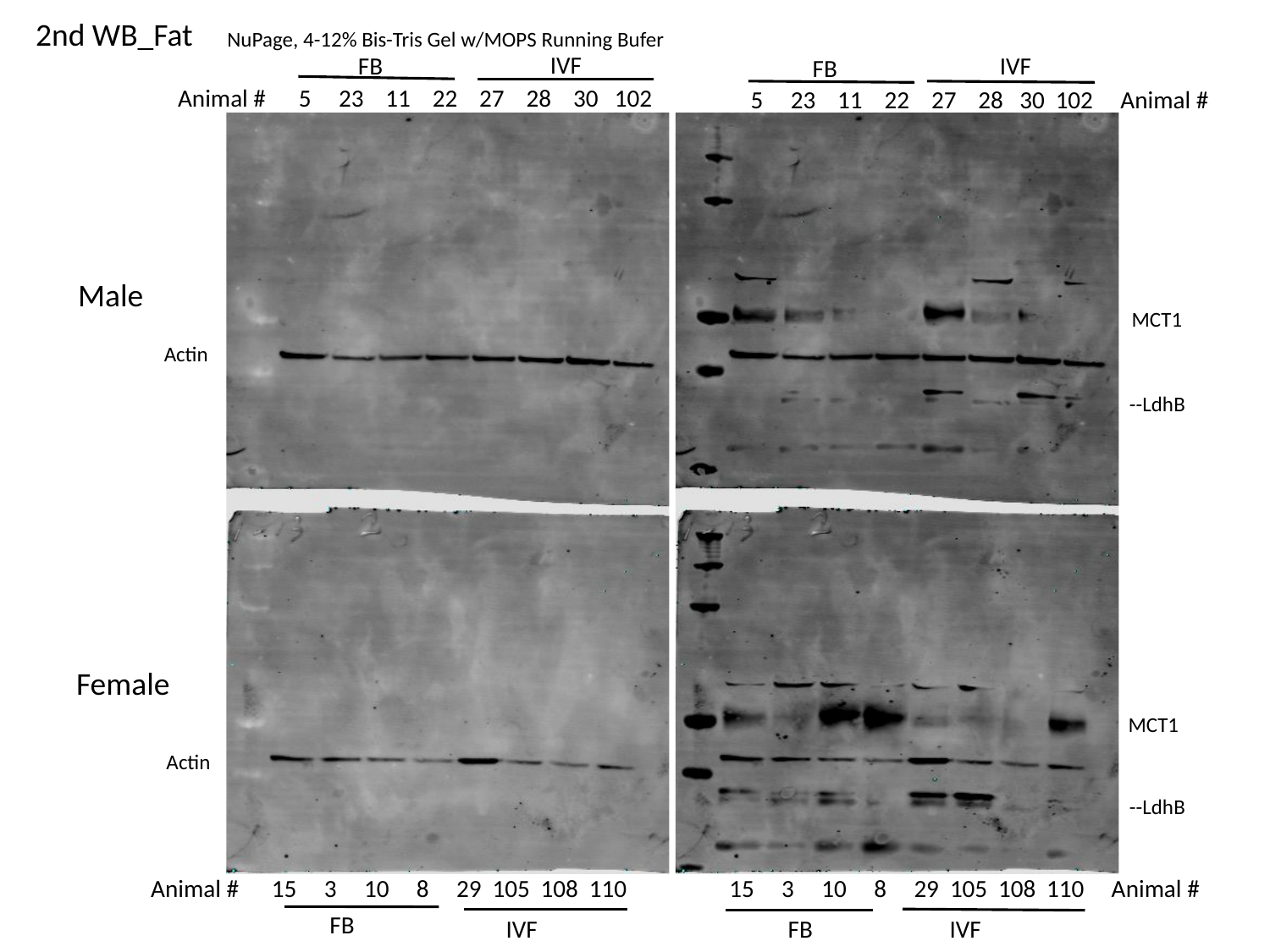

2nd WB_Fat
NuPage, 4-12% Bis-Tris Gel w/MOPS Running Bufer
IVF
FB
IVF
FB
Animal # 5 23 11 22 27 28 30 102
5 23 11 22 27 28 30 102 Animal #
Male
MCT1
Actin
--LdhB
Female
MCT1
Actin
--LdhB
Animal # 15 3 10 8 29 105 108 110
15 3 10 8 29 105 108 110 Animal #
FB
IVF
IVF
FB

## Slide 3
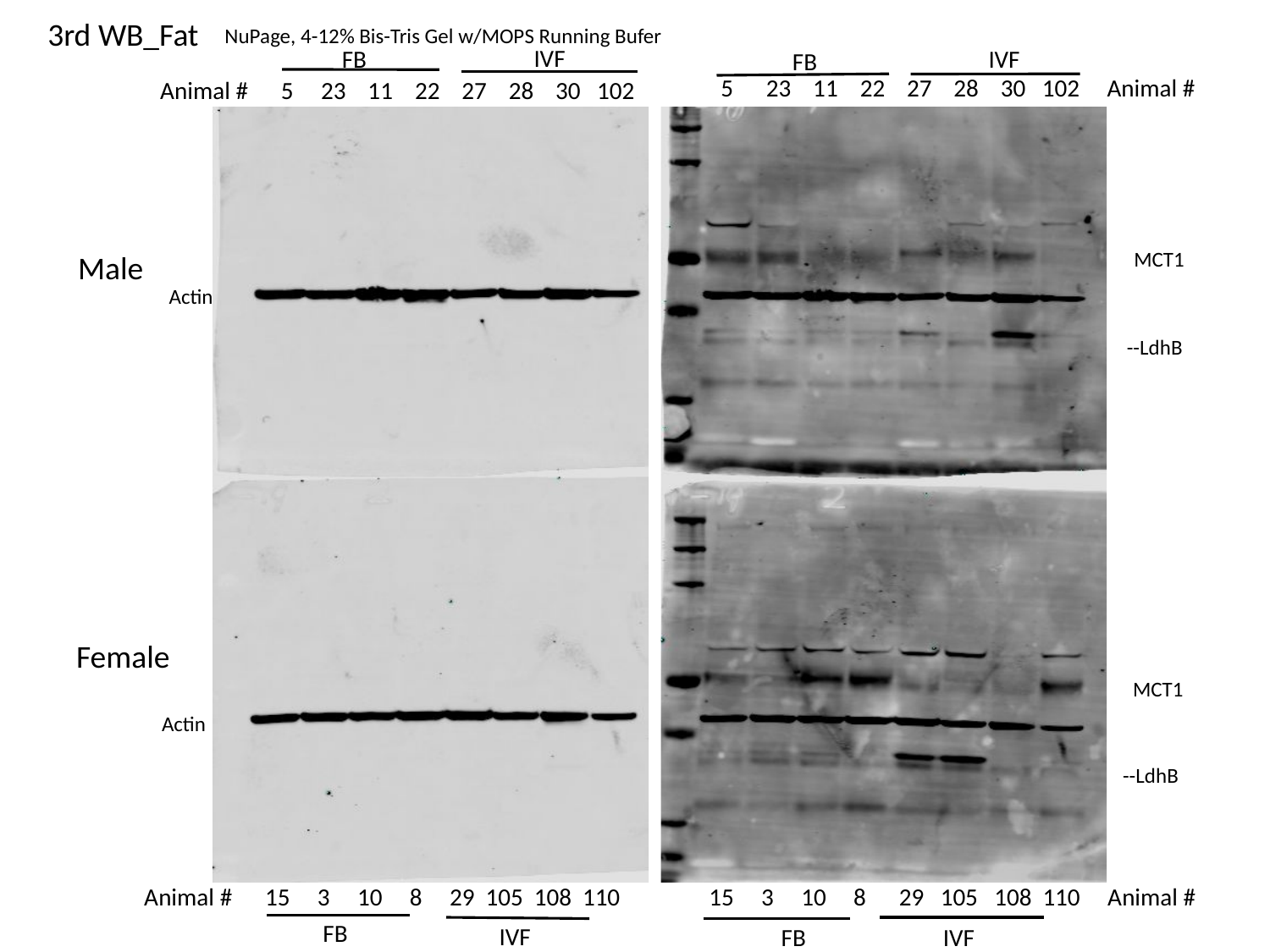

3rd WB_Fat
NuPage, 4-12% Bis-Tris Gel w/MOPS Running Bufer
IVF
FB
IVF
FB
5 23 11 22 27 28 30 102 Animal #
Animal # 5 23 11 22 27 28 30 102
MCT1
Male
Actin
--LdhB
Female
MCT1
Actin
--LdhB
Animal # 15 3 10 8 29 105 108 110
15 3 10 8 29 105 108 110 Animal #
FB
IVF
IVF
FB

## Slide 4
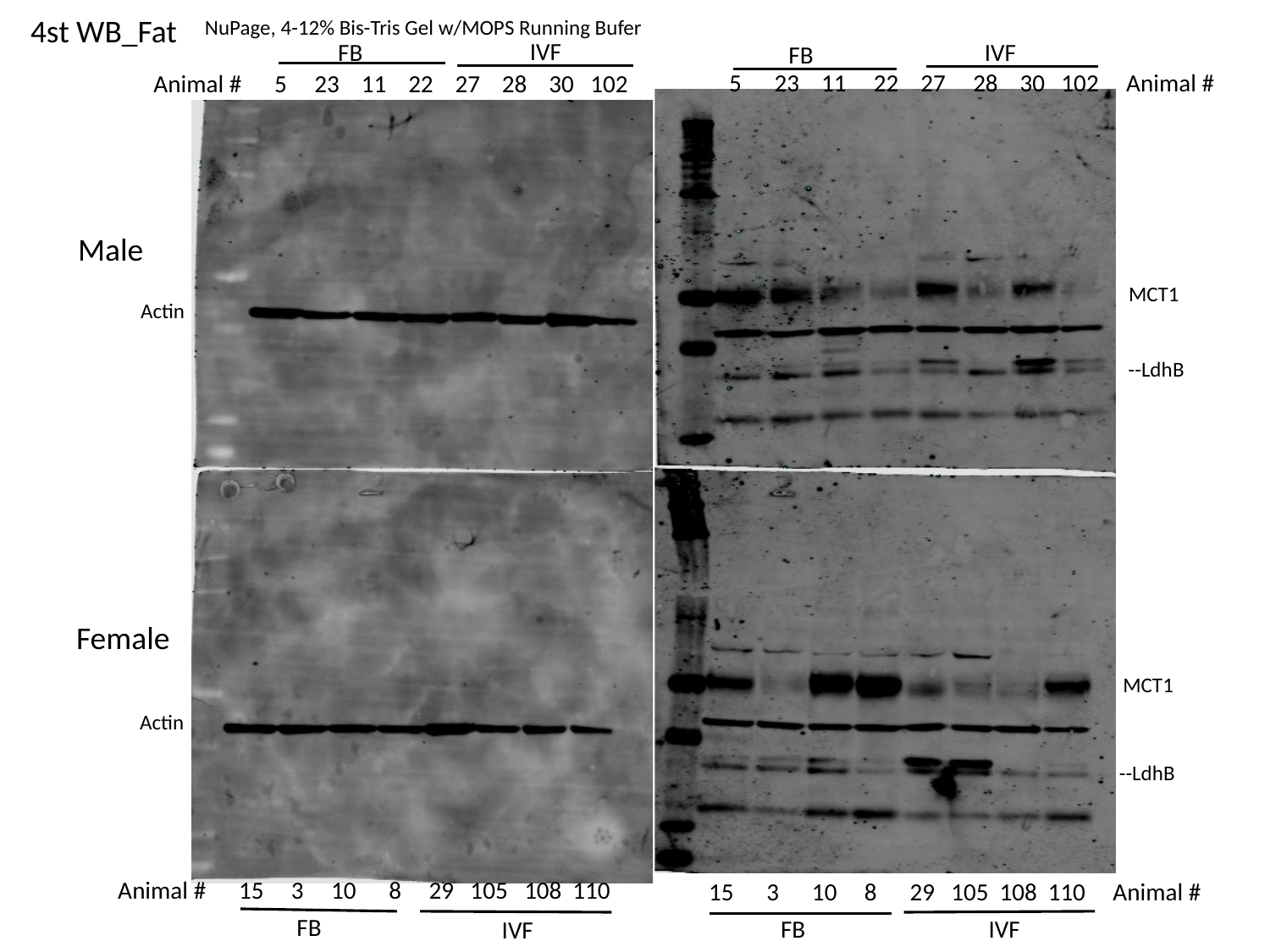

4st WB_Fat
NuPage, 4-12% Bis-Tris Gel w/MOPS Running Bufer
IVF
FB
IVF
FB
5 23 11 22 27 28 30 102 Animal #
Animal # 5 23 11 22 27 28 30 102
Male
MCT1
Actin
--LdhB
Female
MCT1
Actin
--LdhB
Animal # 15 3 10 8 29 105 108 110
15 3 10 8 29 105 108 110 Animal #
FB
FB
IVF
IVF
